# Entropic Probability and Context States

**DOI:** 10.3390/e27020187

**Published:** 2025-02-12

**Authors:** Benjamin Schumacher, Michael D. Westmoreland

**Affiliations:** 1 Department of Physics, Kenyon College, Gambier, OH 43022, USA; 2 Department of Mathematics, Denison University, Granville, OH 43023, USA; westmoreland@denison.edu

**Keywords:** axiomatic thermodynamics, information, entropy, Maxwell’s demon, probability

## Abstract

In a previous paper, we introduced an axiomatic system for information thermodynamics, deriving an entropy function that includes both thermodynamic and information components. From this function, we derived an entropic probability distribution for certain uniform collections of states. Here, we extend the concept of entropic probability to more general collections, augmenting the states by reservoir and context states. This leads to an abstract concept of free energy and establishes a relation between free energy, information erasure, and generalized work.

## 1. Introduction

In [1], we developed an axiomatic system for thermodynamics that incorporated information as a fundamental concept. This system was inspired by previous axiomatic approaches [2,3] and discussions of Maxwell’s demon [4,5]. The basic concept of our system is the *eidostate*, which is a collection of possible states from the point of view of some agent. The axioms imply the existence of additive conserved quantities called *components of content* and an entropy function S that identifies reversible and irreversible processes. The entropy includes both thermodynamic and information components.

One of the surprising things about this axiomatic system is that despite the absence of probabilistic ideas in the axioms, a concept of probability emerges from the entropy S. If state *e* is an element of a uniform eidostate *E*, then we can define

(1) $$P\left(e|E\right)=\frac{2^{S(e)}}{2^{S(E)}}.$$

States in *E* with higher entropy are assigned higher probability. As we will review below, this distribution has a uniquely simple relationship to the entropies of the individual states and the overall eidostate *E*.

The emergence of an entropic probability distribution motivates us to ask several questions. Can this idea be extended beyond uniform eidostates? Can we interpret an arbitrary probability distribution over a set of states as an entropic distribution within a wider context? What does the entropic probability tell us about probabilistic processes affecting the states within our axiomatic system? In this paper, we will address these questions.

## 2. Axiomatic Information Thermodynamics

In physics, a formal axiomatic system can serve several purposes. It can serve to ensure consistent reasoning with unfamiliar or counter-intuitive mathematical objects. Furthermore, it may clarify the underlying concepts of a subject and their relations. This in turn can help to understand how widely, and under what circumstances, those concepts may apply to the physical world. Here, we review the system in [1] and describe the ideas represented by the definitions and axioms.

Our system aims to capture how an agent with finite knowledge might describe states and processes in the external world. The set of *states* is denoted S, but the agent’s knowledge is represented by an *eidostate*, which is a finite collection of *possible* states in S. (The term *eidostate* comes from the Greek *eidos*, meaning *to see*.) Thus, the agent’s knowledge of the state may be incomplete. No probability distribution is assumed a priori for the eidostate; it is a simple enumeration of possibilities. The set of eidostates is called E. (Without too much confusion, we may identify a state a∈S with the singleton eidostate {a}∈E so that S can be regarded as a subset of E.)

Eidostates may be combined by the operation +, which simply represents the Cartesian product of the sets. Thus, if *A* and *B* are distinct eidostates, the combination A+B is not the same as B+A. Two eidostates *A* and $A^{\prime }$ are similar ($A∼A^{\prime }$) if they are formed by the same Cartesian factors perhaps put together in a different way. Our first axiom is

**Axiom** **1** (Eidostates). E is a collection of sets called *eidostates* such that

(a) Every A∈E is a finite non-empty set with a finite prime Cartesian factorization.
(b) A+B∈E if and only if A,B∈E.
(c) Every non-empty subset of an eidostate is also an eidostate.

The agent may control state transformations, including those that involve the acquisition, use, or deletion of information. This produces a relation → on E. We interpret A→B to mean that there exists a dynamical evolution that transforms *A* into *B*, perhaps in the presence of some other “apparatus” states that undergo no net change. We assume that any simple rearrangement of an eidostate’s components is always possible, that transformations can be composed in succession, and so on:

**Axiom** **2** (Processes). Let eidostates A,B,C∈E, and s∈S.

(a) If A∼B, then A→B.
(b) If A→B and B→C, then A→C.
(c) If A→B, then A+C→B+C.
(d) If A+s→B+s, then A→B.

Formally, a *process* is a pair $\langle A,B\rangle$ of eidostates, and it is said to be possible if either A→B or B→A. An eidostate *A* is *uniform* if, for all a,b∈A, $\langle a,b\rangle$ is possible. Uniform eidostates, as we will see, have particularly tractable properties. The existence of *non*-uniform eidostates is neither forbidden nor guaranteed by the axioms, and there are models both with them and without them.

Consider a non-deterministic process in which initial state *a* might evolve into either $b_{1}$ or $b_{2}$. We describe this as a deterministic process on eidostates (that is, $\left\{a\right\}\to \left\{b_{1},b_{2}\right\}$). The principle underlying the “Second Law” in our axiomatic system is simple to state. Given a collection *A* of possible states, no process can deterministically eliminate one or more of the elements of *A* without any other effect. Possibilities cannot simply be “deleted” by the agent. That is,

**Axiom** **3.** If A,B∈E and *B* is a proper subset of *A*, then A↛B.

To borrow an example from the next section, suppose the agent does not know whether a coin is showing “heads” (*h*) or “tails” (*t*). This might be represented by the eidostate {h,t}. The agent might examine the coin to discover its actual state, but this is not a *deterministic* process, and it might end up in either *h* or *t*. To *guarantee* that the coin is *h*, the agent will need to intervene in the coin’s state, which in principle may involve changes to other states.

On the other hand, it is reasonable to assume the existence of certain “conditional” processes:

**Axiom** **4** (Conditional processes).

(a) Suppose $A,A^{\prime }\in E$ and b∈S. If A→b and $A^{\prime }\subseteq A$ then $A^{\prime }\to b$.
(b) Suppose *A* and *B* are uniform eidostates that are each disjoint unions of eidostates: $A=A_{1}\cup A_{2}$ and $B=B_{1}\cup B_{2}$. If $A_{1}\to B_{1}$ and $A_{2}\to B_{2}$ then A→B.

We now introduce information concepts into the system. This is based on the idea of a *record state*. State *r* is a record state if there exists another state such that a+r↔a. That is, a *particular* record state may be freely created or deleted. An information state is an eidostate containing only record states; the set of these is called I. A bit state $I_{b}$ is an information state with exactly two elements, and a bit process is a process of the form $\langle r,I_{b}\rangle$. Then, we have

**Axiom** **5** (Information). There exist a bit state and a possible bit process.

Devices such as Maxwell’s demon accomplish state transformations by acquiring information. We accommodate these into our theory via a new axiom:

**Axiom** **6** (Demons). Suppose a,b∈S and J∈I such that a→b+J.

(a) There exists I∈I such that b→a+I.
(b) For any I∈I, either a→b+I or b+I→a.

The first clause asserts that what one demon can do (transforming *a* into *b* by acquiring the information in *J*), another demon can undo (transforming *b* into *a* by acquiring information in *I*). The second clause envisions nearly reversible demons.

Two more axioms serve more technical purposes. The first regularizes the → relation by its asymptotic behavior:

**Axiom** **7** (Stability). Suppose A,B∈E and J∈I. If nA→nB+J for arbitrarily large values of *n*, then A→B.

The second posits the existence of states in which conserved quantities may be stored—e.g., the height of a weight used for storing energy. We have

**Axiom** **8** (Mechanical states). There exists a subset M⊆S of *mechanical states* such that

(a) If l,m∈M, then l+m∈M.
(b) For l,m∈M, if l→m then m→l.

(This axiom is identical to one posited by Giles [2], whose system was a model for our own.) Note that the axiom does not assert that M is non-empty; it is perfectly consistent to have a model for the axioms with no mechanical states.

We introduce one additional axiom that allows us to relate uniform eidostates to singleton states. Consider, for instance, a situation in which a one-particle “Szilard” gas is confined to a volume. This situation corresponds to a single state $s_{0}$ (as in the first part of Figure 2 below). Now, we introduce a partition so that the gas particle might be either in one side or the other (that is, state $s_{a}$ or $s_{b}$). If we remove the partition, the original state $s_{0}$ is restored. Thus, $a↔\{a_{1},a_{2}\}$. Considering many such examples, we arrive at our final axiom:

**Axiom** **9** (State equivalence). If *E* is a uniform eidostate, then there exist states e,x,y∈S such that x→y and E+x↔e+y.

The system yields some striking results. For instance, we can establish the existence of *components of content*, which are numerical quantities that are conserved in every possible process. A component of content *Q* is a real-valued additive function on the set of states S. (Additive in this context means that Q(a+b)=Q(a)+Q(b).) In a uniform eidostate *E*, every element has the same values of all components of content, so we can without ambiguity refer to the value Q(E). The set of uniform eidostates is denoted U. This set includes all singleton states in S, all information states in I and so forth, and it is closed under the + operation.

Perhaps the most far-reaching theorem established by the axioms is the following (Theorem 8 in [1]):

**Theorem** **1.** *There exist an entropy function S and a set of components of content Q on U with the following properties:*

*(a)*  *For any E,F∈U, S(E+F)=S(E)+S(F).*
*(b)*  *For any E,F∈U and component of content Q, Q(E+F)=Q(E)+Q(F).*
*(c)*  *For any E,F∈U, E→F if and only if S(E)≤S(F) and Q(E)=Q(F) for every component of content Q.*
*(d)*  *S(m)=0 for all m∈M.*

On uniform eidostates, the entropy function S is completely determined (up to a non-mechanical component of content) by the → relations among the eidostates.

## 3. Coin-and-Box Model

Now that we have described our system in some detail, we will introduce a very simple model of the axioms. None of our later results depend on this model, but a definite example will be convenient for explanatory purposes. Our theory deals with configurations of coins and boxes; the states are arrangements of coins, memory records, and closed boxes containing coins. We have the following:

- *Coin states*, which can be either *h* (heads) or *t* (tails) or combinations of these. It is also convenient to define a *stack state* $s_{n}$ to be a particular combination of *n* coin states *h*: $s_{n}=h+\left(h+\left(h+\cdots \right)\right)$. The *coin value Q* of a compound of coin states is just the total number of coins involved. A finite set *K* of coin states is said to be *Q*-uniform if every element has the same *Q*-value.
- Record states *r*. As the name suggests, these should be interpreted as specific values in some available memory register. The combination of two record states is another record state. Thus, *r*, r+r, r+(r+r), etc., are all distinct record states. Record states are not coins, so Q(r)=0.
- Box states. For any *Q*-uniform set of coin states *C*, there is a sequence of *box states* $b_{n}^{K}$. Intuitively, this represents a kind of closed box containing nQ(K) coins so that $Q\left(b_{n}^{K}\right)=nQ\left(K\right)$. If K={h,t}, then we denote the corresponding “basic” box states by $b_{n}$.

An *eidostate* is any finite, non-empty, *Q*-uniform set of states.

We now must define the relation → among eidostates.

- We assume that similar eidostates can be transformed into each other in accordance with our axioms. As far as the → relation is concerned, we can freely rearrange the “pieces” in a compound eidostate.
- For coin states, h↔t.
- If *r* is a record state, a+r↔a for any *a*. In a similar way, for an *empty* box state, $a+b_{0}^{K}↔a$.
- If *K* is a *Q*-uniform eidostate of coin states, $b_{n}^{K}+K↔b_{n+1}^{K}$.

Now, we add some rules that allow us to extend these to more complex situations and satisfy the axioms. In what follows, *A*, $A^{\prime }$, *B*, etc., are eidostates, and *s* is a state.

**Transitivity.**  If A→B and B→C, then A→C.
**Augmentation.**  If A→B, then A+C→B+C.
**Cancelation.**  If A+s→B+s, then A→B.
**Subset.**  If A→s and $A^{\prime }\subseteq A$, then $A^{\prime }\to s$.
**Disjoint** **union.**  If *A* and *B* are both disjoint unions $A=A_{1}\cup A_{2}$ and $B_{1}\cup B_{2}$, and both $A_{1}\to B_{1}$ and $A_{2}\to B_{2}$, then A→B.

Using these rules, we can prove a lot of → relations. For example, for a basic box state, we have $b_{n}+\left\{h,t\right\}↔b_{n+1}$. From the subset rule, we have $b_{n}+h\to b_{n+1}$ (but not the reverse). Then, we can say

(2) $$b_{n}+h\to b_{n+1}\to b_{n}+\left\{h,t\right\},$$

from which we can conclude (via transitivity and cancelation) that h→{h,t}. The use of a basic box allows us to “randomize” the state of one coin.

Or consider two coin states and distinct record states $r_{0}$ and $r_{1}$. Then

(3) $$h\to h+r_{0}\;and\;t\to t+r_{1}\to h+r_{1},$$

from which can show that $\left\{h,t\right\}\to h+\left\{r_{0},r_{1}\right\}$. That is, we can set an unknown coin state to *h* if we also make a record of which state it is. A pretty similar argument establishes the following:

(4) $$\begin{array}{ccc} \left(h+\left\{r_{0},r_{1}\right\}\right)+b_{n} & \to & \left\{h+r_{0},t+r_{1}\right\}+b_{n}\\ & \to & \left(\left\{h,t\right\}+r_{0}\right)+b_{n}\to \left\{h,t\right\}+b_{n}\to b_{n+1}. \end{array}$$

The bit state $\left\{r_{0},r_{1}\right\}$ can be deleted at the cost of a coin absorbed by the basic box. The basic box is a coin-operated deletion device, and since each step above is reversible, we can also use it to dispense a coin together with a bit state (that is, an unknown bit in a memory register).

These examples help us to clarify an important distinction. What is the difference between the box state $b_{1}$ and the eidostate {h,t}? Could we simply replace all box states $b_{n}^{K}$ with a simple combination K+(K+…) of possible coin eidostates? We cannot, because such a replacement would preclude us from using the subset rule to obtain Equation (2). The whole point of the box state is that the detailed state of its contents is *entirely inaccessible* for determining possible processes. From the point of view of the agent, putting a coin in a box effectively randomizes it.

It is not difficult to show that our model satisfies all of the axioms presented in the the last section with the mechanical states in M identified as coin states. The key idea in the proof is that we can reversibly reduce any eidostate to one with a special form:

(5) $$A↔s_{q}+I_{k},$$

where $s_{q}$ is a stack state of q=Q(A) coins and $I_{k}$ is an *information state* containing *k* possible record states. (This is true because all box states can be reversibly “emptied”, and any *Q*-uniform eidostate of coin states can be reversibly transformed into a stack state and an information state, as in Equation (5)). Relations between eidostates are thus reduced to relations between states of this form. We note that the coin value *q* is conserved in every → relation, and no relation allows us to decrease the value of *k*. In our model, there is just one independent component of content (*Q* itself), and the entropy function is S(A)=logk. (We use base-2 logarithms throughout).

## 4. The Entropy Formula and Entropic Probability

Now, let us return to the general axiomatic system. One general result of [1] is a formula for computing the entropy of a uniform eidostate *E* in terms of the entropies of its elements *e*. This is

(6) $$S\left(E\right)=\log\left(\sum_{e\in E}2^{S(e)}\right).$$

It is this equation that motivates our definition of the entropic probability of *e* within the eidostate *E*:

(7) $$P\left(e|E\right)=\frac{2^{S(e)}}{2^{S(E)}}.$$

Then, P(e|E)≥0 and the probabilities sum over *E* to 1. As we have mentioned, the entropy function S may not be quite unique; nevertheless, two different admissible entropy functions lead to the *same* entropic probability distribution. Even better, our definition gives us a very suggestive formula for the entropy of *E*:

(8) $$\begin{array}{ccc} S(E) & = & \sum_{e\in E}P\left(e|E\right)S\left(e\right)-\sum_{e\in E}P\left(e|E\right)\log P\left(e|E\right) \end{array}$$

(9) $$\begin{array}{ccc} & = & \langle S(a)\rangle +H\left(\vec{P}\right), \end{array}$$

where the mean 〈⋯〉 is taken with respect to the entropic probability, and $H(\vec{P})$ is the Shannon entropy of the distribution *P* [6,7].

Equation (9) is very special. If we choose an arbitrary distribution (say $P^{\prime }\left(e|E\right)$), then with respect to this probability, we find

(10) $$S\left(E\right)\geq {\langle S(a)\rangle }_{\;\;P^{\prime }}+H\left({\vec{P}}^{\prime }\right),$$

with equality if and only if $P^{\prime }$ is the entropic distribution [7]. Therefore, we might *define* the entropic probability to be the distribution that maximizes the sum of average state entropy and Shannon entropy—a kind of “maximum entropy” characterization.

How is the entropic probability related to other familiar probability concepts? To quote [1],

Every formal basis for probability emphasizes a distinct idea about it. In Kolmogorov’s axioms [8], probability is simply a measure on a sample space. High-measure subsets are more probable. In the Bayesian approach of Cox [9], probability is a rational measure of confidence in a proposition. Propositions in which a rational agent is more confident are also more probable. Laplace’s early discussion [10] is based on symmetry. Symmetrically equivalent events—two different orderings of a shuffled deck of cards, for instance—are equally probable. (Zurek [11] has used a similar principle of “envariance” to discuss the origin of quantum probabilities.) In algorithmic information theory [7], the algorithmic probability of a bit string is related to its complexity. Simpler bit strings are more probable.

We may remark further that axiom systems like those of Kolmogorov or Cox do not assign actual probabilities in any situation; they simply enforce rules that any such assignment must satisfy. Entropic probability is an actual assignment of a particular distribution, which is determined by objective facts about state transformations.

## 5. Uniformization

A unique entropic probability rule arises from our → relations among eidostates, which in the real world might summarize empirical data about possible state transformations. But so far, this entropic probability distribution P(e|E) is only defined within a uniform eidostate *E*.

In part, this makes sense. An eidostate represents represents the knowledge of an agent—i.e., that the state must be one of those included in the set. This is the knowledge upon which the agent will assign probabilities, which is why we have indicated the eidostate *E* as the *condition* for the distribution. Furthermore, these might be the only eidostates, since the axioms themselves do not guarantee that any non-uniform eidostates exist. (Some models of the axioms have them, and some do not.) But can we generalize the probabilities to distributions over non-uniform collections of states?

Suppose $A=\{a,a^{\prime },\ldots \}$ is a finite set of states that is possibly not uniform. Then, we say that *A* is *uniformizable* if there exists a uniform eidostate $\hat{A}=\left\{a+m_{a},a^{\prime }+m_{a^{\prime }},\ldots \right\}$, where the states $m_{a}$ are mechanical states in M. The idea is that the states in *A*, which vary in their components of content, can be extended by mechanical states that “even out” these variations. Since $\hat{A}$ is uniform, then $a+m_{a}⇌a^{\prime }+m_{a^{\prime }}$ for any $a,a^{\prime }\in A$. The abstract process $\langle a,a^{\prime }\rangle$ is said to be *adiabatically possible* [2]. Mechanical states have $S(m_{a})=0$, so the entropy of the extended $\hat{A}$ is just

(11) $$S\left(\hat{A}\right)=\log\left(\sum_{a\in A}2^{S(a)}\right),$$

which is independent of our choice of the uniformizing mechanical states.

What is the significance of this entropy? Suppose *A* and *B* are not themselves uniform, but their union A∪B is uniformizable. Then, we may construct uniform eidostates $\hat{A}=\left\{a+m_{a},\ldots \right\}$ and $\hat{B}=\left\{b+m_{b},\ldots \right\}$ such that either $\hat{A}\to \hat{B}$ or $\hat{B}\to \hat{A}$, depending on whether $S\left(\hat{A}\right)\geq S\left(\hat{B}\right)$ or the reverse. In short, the entropies of the extended eidostates determine whether the set of states *A* can be turned into the set *B if* we imagine that these states can be augmented by mechanical states, embedding them in a larger, uniform context.

Given the entropy of the extended state, we can define

(12) $$P\left(a|A\right)=P\left(a+m_{a}|\hat{A}\right)=\frac{2^{S(a)}}{2^{S(\hat{A})}}.$$

This extends the entropic probability to the uniformizable set *A*.

Let us consider an example from our coin-and-box model. We start out with the non-uniform set $B=\{b_{n},b_{n+1}\}$. These two basic box states have different numbers of coins. But we can uniformize this set by adding stack states, so that $\hat{B}=\left\{b_{n}+s_{m+1},b_{n+1}+s_{m}\right\}$ is a uniform eidostate. The entropy of a basic box state is $S(b_{n})=n$, so we have

(13) $$S\left(\hat{B}\right)=\log\left(2^{n}+2^{n+1}\right)=\log\left(3\cdot 2^{n}\right)=n+\log3.$$

The entropic probabilities are thus

(14) $$P\left(b_{n}|B\right)=\frac{1}{3}\;\mathrm{and}\;P\left(b_{n+1}|B\right)=\frac{2}{3}.$$

## 6. Reservoir States

So far, we have uniformized a non-uniform set *A* by augmenting its elements with mechanical states, which act as a sort of “reservoir” of components of content. These mechanical states have no entropy of their own. But we can also consider a procedure in which the augmenting states act more like the states of a thermal reservoir in conventional thermodynamics.

We begin with a mechanical state μ, and posit a sequence of *reservoir states* $\theta_{n}$, which have the following properties.

- For any *n*, $\theta_{n}+\mu \to \theta_{n+1}$.
- $\theta_{k}+\theta_{l}↔\theta_{m}+\theta_{n}$ if and only if k+l=m+n.

The reservoir states $\theta_{n}$ form a ladder. We can ascend one rung in the ladder by “dissolving” the mechanical state μ into the reservoir. If we have more than one reservoir state, we can ascend one ladder provided we descend another by the same number of rungs.

For any *n* and *m*, we have that $S\left(\theta_{n}\right)+S\left(\theta_{m+1}\right)=S\left(\theta_{n+1}\right)+S\left(\theta_{m}\right)$, so that

(15) $$\sigma =S\left(\theta_{n+1}\right)-S\left(\theta_{n}\right)=S\left(\theta_{m+1}\right)-S\left(\theta_{m}\right)$$

is a non-negative constant for the particular sequence of reservoir states. This sequence $\left\{\theta_{n}\right\}$ is characterized by the state μ and the entropy increment σ. Note that we can write $S\left(\theta_{n}\right)=n\sigma +S_{0}$, where $S_{0}=S\left(\theta_{0}\right)$.

For example, in our coin-and-box model, the basic box states $b_{n}$ act as a sequence of reservoir states with a mechanical (coin) state μ=h and an entropy increment σ=log2=1. The more general box states $b_{n}^{K}$ form a reservoir state sequence with $\mu =s_{q}$ and σ=logk, where q=Q(K) and *k* is the number of states in *K*. For each of these box-state reservoir sequences, $S_{0}=S\left(\theta_{0}\right)=0$.

One particular type of reservoir is a *mechanical* reservoir consisting of the states μ, μ+μ, μ+(μ+μ), etc. We denote the *n*th such state by $\mu_{n}$. For the $\mu_{n}$ reservoir states, σ=0. If we have a finite set of states $A=\{a,a^{\prime },\ldots \}$ that can be uniformized by the addition of the $\mu_{n}$ states, they can also be uniformized by a corresponding set of non-mechanical reservoir states $\theta_{n}$:

(16) $$\hat{A}=\left\{a+\theta_{n_{a}},a^{\prime }+\theta_{n_{a^{\prime }}},\ldots \right\}.$$

As before, we can find the entropy of this uniform eidostate and define entropic probabilities. But the θ reservoir states now contribute to the entropy and affect the probabilities.

First, the entropy:

(17) $$S\left(\hat{A}\right)=\log\left(\sum_{a\in A}2^{S\left(a\right)+S\left(\theta_{n_{a}}\right)}\right)=S_{0}+\log\left(\sum_{a\in A}2^{S(a)}2^{n_{a}\sigma }\right).$$

The entropic probability—which now depends on the choice of reservoir states—is

(18) $$P_{\theta }\left(a|A\right)=P\left(a+\theta_{n_{a}}|\hat{A}\right)=\frac{2^{S\left(a\right)+n_{a}\sigma }}{\left(\sum_{a\in A}2^{S\left(a\right)+n_{a}\sigma }\right)}.$$

The reservoir states affect the relative probabilities of the states. For example, suppose $S\left(a\right)=S\left(a^{\prime }\right)$ for a pair of states in *A*. We might naively think that these states would end up with the same entropic probability, as they would if we uniformized *A* by mechanical states. But since we are uniformizing using the θ reservoir states, it may be that $\theta_{n_{a}}$ and $\theta_{n_{a^{\prime }}}$ have different entropies. Then, the ratio of the probabilities is

(19) $$\frac{P_{\theta }\left(a|A\right)}{P_{\theta }\left(a^{\prime }|A\right)}=\frac{2^{n_{a}\sigma }}{2^{n_{a^{\prime }}\sigma }}=2^{(n_{a}-n_{a^{\prime }})\sigma },$$

which may be very different from 1.

Again, let us consider our coin-and-box model. We begin with the non-uniform set A={h,t,h+t}. Each of these states has the same entropy S, that is, zero. We choose to uniformize using basic box states $b_{n}$. For instance, we might have

(20) $$\hat{A}=\left\{h+b_{1},t+b_{1},\left(h+t\right)+b_{0}\right\}.$$

Recalling that σ=1, the entropy is

(21) $$S\left(\hat{A}\right)=\log\left(2^{1}+2^{1}+2^{0}\right)=\log5.$$

This yields probabilities

(22) $$P_{b}\left(h|A\right)=\frac{2}{5}\;P_{b}\left(t|A\right)=\frac{2}{5}\;P_{b}\left(h+t|A\right)=\frac{1}{5}.$$

**Figure 1:**
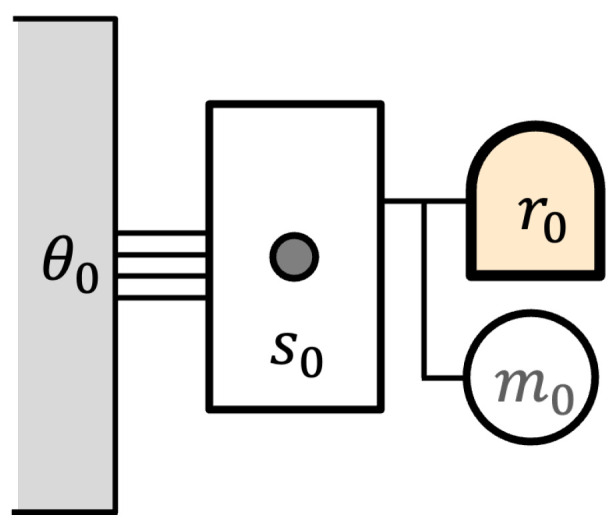
A simple Maxwell’s demon.

As an illustration of these ideas, consider the version of Maxwell’s demon shown in Figure 1.

The demon is a reversible computer with an initial memory state $r_{0}$. It is equipped with a reversible battery for storing energy, initially in mechanical state $m_{0}$. The demon interacts with a one-particle “Szilard” gas, in which the single particle can move freely within its volume (state $s_{0}$). The gas is maintained in thermal equilibrium with a heat reservoir whose initial state is $\theta_{0}$. We might denote the overall initial state by $\left(\left(r_{0}+m_{0}\right)+s_{0}\right)+\theta_{0}$.

**Figure 2:**
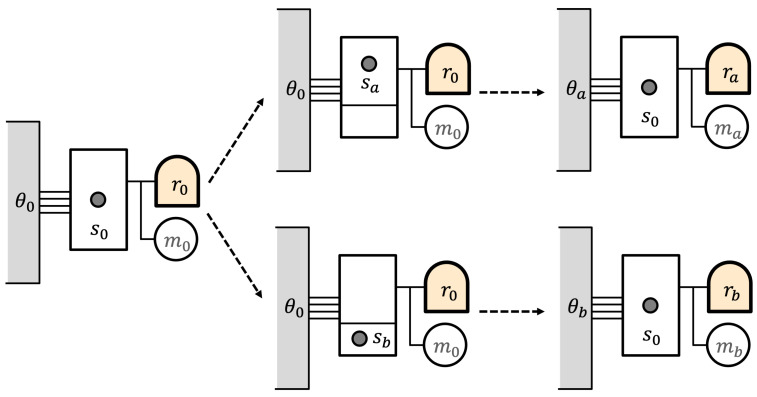
Extraction of work by dividing gas enclosure into unequal volumes.

Now, the demon introduces a partition into the gas, separating the enclosure into unequal subvolumes, as in Figure 2. The two resulting states are $s_{a}$ and $s_{b}$, which are not equally probable. The probabilities here are entropic probabilities due to the difference in entropy of $s_{a}$ and $s_{b}$.

Now, the demon records the location of the particle in its memory and uses this to control the isothermal expansion of the one-particle gas. The work is stored in the battery. At the end of this process, the demon retains its memory record, and the battery is in one of two mechanical states $m_{a}$ and $m_{b}$. The gas is again in state $s_{0}$. But different amounts of heat have been extracted from the reservoir during the expansion, so the reservoir has two different states $\theta_{a}$ and $\theta_{b}$.

The overall final eidostate might be represented as

(23) $$F=\{\left(\left(r_{a}+m_{a}\right)+s_{0}\right)+\theta_{a},\left(\left(r_{b}+m_{b}\right)+s_{0}\right)+\theta_{b}\}.$$

The states of the demon and the gas, $\left(r_{a}+m_{a}\right)+s_{0}$ and $\left(r_{b}+m_{b}\right)+s_{0}$, have different energies and the same entropy. It is the reservoir states $\theta_{a}$ and $\theta_{b}$ that (1) make *F* uniform (constant energy) and (2) introduce the entropy differences leading to different entropic probabilities for the two states.

A conventional view would suppose that the unequal probabilities for the two final demon states comes from their history—that is, that the probabilities are inherited from the unequal partition of the gas. In the entropic view, the unequal probabilities are due to differences in the *environment* of the demon, which is represented by the different reservoir states $\theta_{a}$ and $\theta_{b}$. The environment, in effect, serves as the “memory” of the history of the process.

## 7. Context States

When we uniformize a non-uniform *A* by means of a sequence of reservoir states, the reservoir states affect the entropic probabilities. We can use this idea more generally.

For example, in our coin-and-box model, suppose we flip a coin but do not know how it lands. This might be represented by the eidostate F={h,t}. Without further information, we would assign the coin states equal probability 1/2, which is the simple entropic probability. But suppose we have additional information about the situation that would lead us to assign probabilities 1/3 and 2/3 to the coin states. This additional information—this *context*—must be reflected in the eidostate. The example in Equation (14) tells us that this does the job:

(24) $$\hat{F}=\left\{h+\left(b_{n}+s_{m+1}\right),t+\left(b_{n+1}+s_{m}\right)\right\}.$$

The extended coin-flip state $\hat{F}$ includes extra context so that the entropic probability reflects our additional information.

In general, we can adjust our entropic probabilities by incorporating *context states*. Suppose we have a uniform eidostate $E=\{e_{1},e_{2},\ldots \}$, but we wish to *specify* a particular non-entropic distribution $p_{k}$ over these states. Then, for each $e_{k}$, we introduce eidostates $C_{k}$, leading to an extended eidostate

(25) $$\hat{E}=\underset{k}{⋃}\left(e_{k}+C_{k}\right),$$

which we assume is uniform. The $C_{k}$ values are the context states. Our challenge is to find a set of context states so that the entropic probability in $\hat{E}$ equals the desired distribution $p_{k}$.

We cannot always do this exactly, but we can always approximate it as closely as we like. First, we note that we can always choose our context eidostates to be information states. The information state $I_{n}$ containing *n* record states has entropy logn. Now, for each *k*, we closely approximate the ratio $p_{k}/2^{S(e_{k})}$ by a rational number; and since there are finitely many of these numbers, we can represent them using a common denominator. In our approximation,

(26) $$\frac{p_{k}}{2^{S(e_{k})}}=\frac{n_{k}}{N}.$$

Now, choose $C_{k}=I_{n_{k}}$ for each *k*. The entropy of $\hat{E}$ becomes

(27) $$\begin{array}{ccc} S(\hat{E}) & = & \log\left(\sum_{k}2^{S(e_{k}+\log n_{k})}\right)\\ & = & \log\left(\sum_{k}n_{k}2^{S(e_{k})}\right)=\log\left(\sum_{k}p_{k}N\right)=\log N. \end{array}$$

From this, we find that the entropic probability is

(28) $$P\left(e_{k}+I_{k}|\hat{E}\right)=\frac{n_{k}2^{S(e_{k})}}{N}=p_{k},$$

as desired.

We find, therefore, that the introduction of context states $C_{k}$ allows us to “tune” the entropic probability to approximate any distribution $p_{k}$ that we like. This is more than a trick. The distribution $p_{k}$ represents additional implicit information (beyond the mere list of states $E=\{e_{k}\}$), and such additional information must have a physical representation. The context states are that representation.

## 8. Free Energy

The tools we have developed can lead to some interesting places. Suppose we have two sets of states, $A=\{a_{i}\}$ and $B=\{b_{j}\}$, endowed with a priori probability distributions $p_{i}$ and $q_{j}$, respectively. We wish to know when the states in *A* can be turned into the states in *B*, which are perhaps augmented by reservoir states. That is, we wish to know when $\hat{A}\to \hat{B}$.

We suppose we have a mechanical state μ, leading to a ladder of mechanical reservoir states $\mu_{n}=\mu +\left(\mu +\ldots \right)$. The mechanical state μ is non-trivial in the the sense that s+μ↛s for any *s*. This means that there is a component of content *Q* such that Q(μ)≠0. The set A∪B can be uniformized by augmenting the $a_{i}$ and $b_{j}$ states by $\mu_{n}$ mechanical reservoir states.

However, we still need to realize the $p_{i}$ and $q_{j}$ probabilities. We do this by introducing as context states a corresponding ladder of reservoir states $\theta_{n}$ such that $\sigma =S\left(\theta_{n+1}\right)-S\left(\theta_{n}\right)$ is very small. Essentially, we assume that the reservoir states are “fine-grained” enough that we can approximate any positive number by $2^{n\sigma }$ for some positive or negative integer *n*. Then, if we augment the $a_{i}$ and $b_{j}$ states by combinations of $\mu_{n}$ and $\theta_{n}$ states, we can uniformize A∪B and also tune the entropic probabilities to match the a priori $p_{i}$ and $q_{j}$. The final overall uniform eidostate is

(29) $$\{a_{i}+\left(\mu_{l_{i}}+\theta_{k_{i}}\right),b_{j}+\left(\mu_{h_{j}}+\theta_{n_{j}}\right)\},$$

for integers $l_{i}$, $k_{i}$, $h_{j}$ and $n_{j}$. The uniformized $\hat{A}$ and $\hat{B}$ eidostates are subsets of this and thus are themselves uniform eidostates. The entropic probabilities have been adjusted so that

(30) $$p_{i}=\frac{2^{S\left(a_{i}\right)+k_{i}\sigma +S\left(\theta_{0}\right)}}{2^{S(\hat{A})}}\;\mathrm{and}\;q_{j}=\frac{2^{S\left(b_{j}\right)+n_{j}\sigma +S\left(\theta_{0}\right)}}{2^{S(\hat{B})}}.$$

We now choose a component of content *Q* such that Q(μ)=ε>0. Since the overall state is is uniform, it must be true that

(31) $$Q\left(a_{i}\right)+l_{i}\varepsilon +k_{i}\varepsilon =Q\left(b_{j}\right)+h_{j}\varepsilon +n_{j}\varepsilon =\mathrm{constant}$$

for all choices of i,j. Of course, if all of these values are the same, we can average them together and obtain

(32) $${\langle Q(a_{i})\rangle }_{\;p}+{\langle l_{i}\rangle }_{\;p}\varepsilon +{\langle k_{i}\rangle }_{\;p}\varepsilon ={\langle Q(b_{j})\rangle }_{\;q}+{\langle h_{j}\rangle }_{\;q}\varepsilon +{\langle n_{j}\rangle }_{\;q}\varepsilon .$$

We can write the average change in the *Q*-value of the mechanical state as

(33) $$\left({\langle h_{j}\rangle }_{\;q}-{\langle l_{i}\rangle }_{\;p}\right)\varepsilon =\left({\langle k_{i}\rangle }_{\;p}-{\langle n_{j}\rangle }_{\;q}\right)\varepsilon +{\langle Q(a_{i})\rangle }_{\;p}-{\langle Q(b_{j})\rangle }_{\;q}.$$

Since all of the states lie within the same uniform eidostate, $\hat{A}\to \hat{B}$ if and only if $S\left(\hat{A}\right)\leq S\left(\hat{B}\right)$—that is,

(34) $$H\left(\vec{p}\right)+{\langle S(a_{i})\rangle }_{\;p}+{\langle k_{i}\rangle }_{\;p}\sigma \leq H\left(\vec{q}\right)+{\langle S(b_{j})\rangle }_{\;q}+{\langle n_{j}\rangle }_{\;q}\sigma .$$

From this, it follows that

(35) $$\left({\langle k_{i}\rangle }_{\;p}-{\langle n_{j}\rangle }_{\;q}\right)\leq \frac{1}{\sigma }\left(H\left(\vec{q}\right)-H\left(\vec{p}\right)+{\langle S(b_{j})\rangle }_{\;q}-{\langle S(a_{i})\rangle }_{\;p}\right).$$

If we substitute this inequality into Equation (33), we obtain

(36) $$\begin{array}{ccc} \left({\langle h_{j}\rangle }_{\;q}-{\langle l_{i}\rangle }_{\;p}\right)\varepsilon -\frac{\varepsilon }{\sigma }\left(H\left(\vec{q}\right)-H\left(\vec{p}\right)\right) & \leq & +\frac{\varepsilon }{\sigma }\left({\langle S(b_{j})\rangle }_{\;q}-{\langle S(a_{i})\rangle }_{\;p}\right)\\ & & -\left({\langle Q(b_{j})\rangle }_{\;q}-{\langle Q(a_{i})\rangle }_{\;p}\right). \end{array}$$

We can obtain insight into this expression as follows. Given the process $\hat{A}\to \hat{B}$,

- $\left({\langle h_{j}\rangle }_{\;q}-{\langle l_{i}\rangle }_{\;p}\right)\varepsilon$ is the average increase in *Q*-value of the mechanical state, which we can call $\langle \Delta Q_{\mu }\rangle$. Intuitively, this might be regarded as the “work” stored in the $\hat{A}\to \hat{B}$ process.
- We can denote the change in the Shannon entropy of the probabilities by $\Delta H=H\left(\vec{q}\right)-H\left(\vec{p}\right)$. Since each $a_{i}$ or $b_{j}$ state could be augmented by a corresponding record state, this is the change in the information entropy of the stored record.
- For each state *a*, we can define the *free energy* $F\left(a\right)=Q\left(a\right)-\frac{\varepsilon }{\sigma }S\left(a\right)$. We call this free “energy”, even though *Q* does not necessarily represent energy, because of the analogy with the familiar expression F=E−TS for the Helmholtz free energy in conventional thermodynamics. The average change in the free energy *F* is
  (37) $$\langle \Delta F\rangle =\left({\langle Q(b_{j})\rangle }_{\;q}-{\langle Q(a_{i})\rangle }_{\;p}\right)-\frac{\varepsilon }{\sigma }\left({\langle S(b_{j})\rangle }_{\;q}-{\langle S(a_{i})\rangle }_{\;p}\right).$$
  The free energy *F* depends on the particular reservoir states $\theta_{n}$ only via the ratio ε/σ. Given this value, $\langle \Delta F\rangle$ depends only on the $a_{i}$ and $b_{j}$ states, together with their a priori probabilities.
- To return to our coin-and-box example, suppose we use the basic box states $b_{n}$ as reservoir states $\theta_{n}$, and we choose the coin number *Q* as our component of content. Then, ε=1 and σ=1, so that the free energy function F(a)=Q(a)−S(a). (If we use different box states $b_{n}^{K}$ as reservoir states, the ratio ε/σ is different.)

With these definitions, Equation (36) becomes
  (38) $$\langle \Delta Q_{\mu }\rangle -\frac{\varepsilon }{\sigma }\Delta H\leq -\langle \Delta F\rangle .$$

Increases in the average stored mechanical work, and decreases in the stored information, must be paid for by a corresponding decrease in the average free energy.

Many useful inferences can be drawn from this. For example, the erasure *Q*-cost of one bit of information in the presence of the θ-reservoir is ε/σ. This cost can be paid from either the mechanical *Q*-reservoir state, the average free energy, or from a combination of these. This amounts to a very general version of Landauer’s principle [12]: one that involves any type of mechanical component of content.

## Data Availability

The original contributions presented in this study are included in the article. Further inquiries can be directed to the corresponding author.
